# Association between lifestyle and musculoskeletal pain: cross-sectional study among 10,000 adults from the general working population

**DOI:** 10.1186/s12891-019-3002-5

**Published:** 2019-12-17

**Authors:** Jéssica Kirsch Micheletti, Rúni Bláfoss, Emil Sundstrup, Hans Bay, Carlos Marcelo Pastre, Lars Louis Andersen

**Affiliations:** 1 National Research Centre for the Working Environment, Lersø Parkalle 105, DK-2100 Copenhagen, Denmark; 2 São Paulo State University (UNESP), 305 Roberto Simonsen, Presidente Prudente, Sao Paulo 19060-900 Brazil; 3 Department of Sports Science and Clinical Biomechanics, SDU Muscle Research Cluster (SMRC), University of Southern Denmark, DK-5250 Odense, Denmark; 4 Department of Physiotherapy, Univ Estadual Paulista (UNESP), 305 Roberto Simonsen, Presidente Prudente, Sao Paulo 19060-900 Brazil; 5 Sport Sciences, Department of Health Science and Technology, Aalborg University, Aalborg, Denmark

**Keywords:** Musculoskeletal diseases, chronic pain, Low back pain, Neck pain, Exercise, health, smoking

## Abstract

**Background:**

Work-related musculoskeletal pain is a major cause of work disability and sickness absence. While pain is a multifactorial phenomenon being influenced by work as well as lifestyle, less is known about the association between specific lifestyle factors and the type of musculoskeletal pain. The aim of the study was to investigate if a dose-response association existed between lifestyle factors and musculoskeletal pain intensity in the low back and neck-shoulder.

**Methods:**

Currently employed wage earners (*N* = 10,427) replied in 2010 to questions about work environment, lifestyle and health. Logistic regression analyses adjusted for various confounders tested the association of alcohol intake, physical activity, fruit and vegetable intake, and smoking (explanatory variables) with low back pain and neck-shoulder pain intensity (outcomes variables, scale 0–9, where ≥4 is high pain).

**Results:**

The minimally adjusted model found that physical activity and fruit and vegetable intake were associated with lower risk of musculoskeletal pain, while smoking was associated with higher risk of musculoskeletal pain. In the fully adjusted model, physical activity ≥5 h per week was associated with lower risk of low back pain and neck-shoulder pain with risk ratios (RR) of 0.95 (95% CI 0.90–1.00) and 0.90 (95% CI 0.82–0.99), respectively. No association was found between alcohol intake and pain.

**Conclusion:**

Being physically active associated with lower risk of having musculoskeletal pain, while smoking habits and healthy eating were associated with higher pain when adjusting for age and gender. Considering the continuously increasing retirement age in many societies, initiatives to promote healthy habits should still be a political priority to help the workers to stay healthy and cope to their work.

## Background

Low back pain and neck-shoulder pain affect between 51 and 90% and 14–71% of people at some point during their lifetime, respectively [1–3], and are the most common symptoms for seeking a physician [4]. Furthermore, low back pain and neck-shoulder pain are considered major public health problems that limit productivity at work and may have enormous medical and economic consequences on individuals, families, communities, industries and societies [5, 6]. In 2013, low back pain and neck-shoulder pain accounted for the third-highest amount of health-care spending in the United States, with an estimated cost of $87.6 billion [7]. Ferguson et al. [8] observed in a data set with nearly 2000 workers in various regions of the United States a prevalence rate of 25% for low back pain lasting at least 1 week. Considering the growing aging population in many societies, musculoskeletal problems are likely to increase, thus necessitating a better understanding of these disorders [9, 10].

Low back pain and neck-shoulder pain are multifactorial disorders related to both individual, physical and psychosocial work environmental factors [11]. In the past years, investigations of risk factors for low back pain and neck-shoulder pain among workers have been conducted in different parts of the world [12–19], and work factors such as hard physical work [20] and prolonged standing [21] have been identified as risk factors for musculoskeletal disorders. However, less is known about the consequences of poor lifestyle, such as low level of physical activity during leisure, smoking, alcohol intake and diet, in relation to low back pain and neck-shoulder pain.

Physical activity and exercise programs are increasingly being promoted and offered in various healthcare systems. A Cochrane review from 2017 [22] indicated that physical activity and exercise is an intervention with few adverse events being effective in decreasing low-back pain severity and physical function, and consequently improving quality of life. Therefore, more research is needed to observe the relationship of lifestyle factors with musculoskeletal pain.

Considering that cigarette smoking is one of the leading causes of premature morbidity and mortality [23], systematic reviews and meta analyses have been attempting to clarify its relationship with musculoskeletal disorders. In a review of association between smoking and low back pain, Stienen et al. concluded that [24] a high number of studies did not observe an association between smoking and low back pain. However, Shiri et al. [25] performed a meta-analysis and observed a stronger association between smoking and low back pain in adolescents than in adults. Few studies regarding the working population were included in this meta-analysis. A recent study from 2017 [26] with a sample of 60 pain-free administrative workers of German universities, showed an increased risk of developing low back pain among the proportion of smokers.

Alcohol intake is another risk factor that needs to be considered. Few reviews have been carried out to investigate the association of alcohol intake with low back pain. In 2000, Leboeuf-Yde [27] performed a systematic review assessing the association between alcohol intake and the risk of developing a new episode of acute low back pain including 9 cross-sectional studies. The authors concluded that alcohol intake is not associated with low back pain. However, 2013 Ferreira et al. [28] performed another systematic review with a broader search strategy than that by Leboeuf-Yde et al. [27]. Twenty-six studies were included, and the results showed that alcohol consumption was associated with low back pain. However, this relation appears only to include complex and chronic low back pain and only generalizable to people with alcohol addiction.

Moreover, healthy lifestyle habits such as consumption of fruit and vegetables have also been investigated, however the number of studies is limited. This habit is considered as a part of healthy lifestyle behavior [29] or optimal lifestyle [30] defined by a combination of lifestyle factors and associated with the prognosis of low back pain [29]. In the study of Bohman et al. [29] with a sample size of 3938 men and 5056 women aged 18–84, an influence of healthy lifestyle behavior was observed on the prognosis of occasional low back pain. The results showed that the healthy lifestyle behavior is associated with decreased low back pain of long duration among women with occasional low back pain.

Altogether, more data is necessary to better understand the association between healthy lifestyle behavior and musculoskeletal disorders in workers. Because the working population is aging in many societies, a healthy lifestyle behavior may be important for staying healthy and cope to the work until high age. Thus, the present study aims to examine the joint association between four lifestyle behavior factors (physical activity, consumption of vegetables and fruits, alcohol intake and smoking) and musculoskeletal pain in the low back and the neck-shoulder among more than 10,000 adults from the general working population in Denmark.

## Methods

### Population

The present study is a cross-sectional study consisting a questionnaire survey about work environment and health in the general working population in Denmark, the Danish Work Environment Cohort Study (DWECS) [31]. The questionnaire was sent out to approximately 20,000 working adults in 2010. A total of 10,605 workers (approximately 53%) replied [32]. In this study, we included only currently employed wage earners from the 2010 round (*n* = 10,427), i.e. excluding self-employed people and people not affiliated with the labour market [33]. Because not all participants filled in all questions, the exact number for each analysis varies.

### Ethical approval

The present study has been notified to and registered by The Danish Data Protection Agency (journal number 2015-57-0074). According to the Danish law, questionnaire- and register-based studies do not need approval by ethical and scientific committees, nor informed consent (Committee System on Biomedical Research Ethics, 2011; The Danish Data Protection Agency, 2008). All data were de-identified and analyzed anonymously.

### Outcome variables

#### Low back pain and neck-shoulder pain intensity

Low back pain and neck-shoulder pain intensity were assessed by replying to the question “Have you experienced any trouble (pain or discomfort) in your (body region) during the last 12 months?” on a scale ranging from 0 to 9, where 0 is no pain and 9 is the worst imaginable pain. For further analyses, back pain was dichotomized into “High pain” (pain intensity ≥4), “No or little pain” (pain intensity 0–3).

### Explanatory variables

#### Physical activity

Physical activity was assessed by the question: “How much time did you on average spend on each of the following leisure-time activities during the last year (including commuting to and from work)?”

It had the following three sub-questions of 1) low, 2) moderate and 3) high-intensity exercise: 1) “Walking, biking or other low-intensity exercise, where you do not get out of breath or sweaty (e.g. Sunday walks or low-intensity gardening)?” 2) “Exercise training, heavy gardening or fast walk/cycling, where you get out of breath and sweaty?” and 3) “Vigorous exercise or competitive sports?”. The response options were: Do not perform this activity; < 2 h per week; 2–4 h per week; or > 4 h per week [34]. These four categories were recoded to 0, 1, 3 or 5 h per week, respectively [34–36]. For the subsequent analysis, the hours of moderate and high-intensity exercise was summed and defined the weekly hours of exercise. As this variable is not strictly linear, we further categorized the sum of exercise into 0, 1–2, 3–4, and ≥ 5 h of moderate to high-intensity exercise per week.

#### Alcohol intake

Alcohol intake was assessed by two questions: (1) “How much alcohol do you drink on average on weekdays (Monday to Thursday)?”, and (2) “How much alcohol do you drink on average Friday, Saturday and Sunday?” The amount of alcohol was considered as number of units per day. One unit = 1 bottle of beer, 1 glass of wine or 2 cl. liquor.

For further analyses, the number of units from the two questions were summed and further grouped into: “0-7 units per week”; “8-14 units per week”; “15-21 units per week”; “>21 units per week”.

#### Smoking status

The smoking status was assessed by the following question: “Do you smoke?”. The response categories were: (1) “Yes, daily”; (2) “Yes, sometimes”; (3) “Used to smoke but not anymore”; (4) “Have never smoked”. For subsequent analyses, the answers were allocated to the following categories: “No, never,” “Ex-smoker” or “Yes”.

Furthermore, the quantity of smoking was assessed by the following question: “If you smoke, how much do you smoke per day on average?” The answers were allocated in the following groups: “0 cigarettes per day”; “1-9 cigarettes per day”; “10-20 cigarettes per day”; “≥ 20 cigarettes per day”.

#### Fruit and vegetable intake

The consumption of fruits and vegetables were assessed by the following question: “How often do you eat fruit, salad / raw food, cooked vegetables - apart from potatoes?”. The response options were: (1) “At least 3 times per day”; (2) “Twice per day”; (3) “1 time per day”; (4) “3-6 times per week”; (5) “1-2 times per week”; (6) “Rarer”. For subsequent analyses, the response categories were divided into: “≤ 2 days per week”; “3-6 days per week”; “Daily”.

### Control variables

Control variables for the analyses in the present study included age (continuous, years) [37], gender (categorical: “male”, “female”) [37], body mass index (BMI) (continuous, kg/m^2^), physical activity at work (categorical, “Standing or walking work with lifting tasks” and “Heavy and fast strenuous work) [38], psychosocial work factors (continuous scale from 0 to 100, single items on influence and emotional demands at work from the Copenhagen Psychosocial Questionnaire) [39], job group (categorical, information about 86 different job groups delivered by Statistics Denmark, e.g. office workers, school teachers, nurses) [33], and chronic disease (categorical) [40]. Chronic disease was based on the question, “Have you ever been informed by a physician that you have or have had one or more of the following conditions?” with the response options being “yes” and “No, never” to the following diseases: Depression, cardiovascular disease, and cancer.

### Statistics

Using logistic regression analyses (PROC GENMOD of SAS version 9.2), the risk for high pain (≥4 on a scale of 0–10) in the low back and neck-shoulder, respectively, was estimated. Risk ratios (RR) and 95% confidence intervals (95% CI) were calculated for high pain as outcome variable. Explanatory mutually adjusted factors were the four lifestyle factors. Model 1 was, besides the explanatory variables (healthy lifestyles), additionally adjusted for age and gender. Model 2 was, besides age and gender, additionally adjusted for BMI, physical activity at work, psychosocial work factors, job group, and chronic disease.

## Results

Table 1 describes the characteristics of the study population. The proportion of female and male gender was 54.3 and 45.7%, respectively. Average age was 43.3 ± 11.7 years, average BMI was 25.4 ± 4.4, and 46.9% had sedentary jobs while 53.1% had jobs with physically demanding job tasks. Low-intensity pain was most frequent in the low back and neck-shoulder region, with a prevalence of 71 and 70% for low back pain and neck-shoulder pain, respectively.

**Table 1:** Demographics, lifestyle, and work-related and health characteristics

	N	Mean	SD	%
Age, years	10,427	43.5	11.7	
BMI (kg.m^−2^)	10,095	25.4	4.4	
Gender				
Men	4762			45.7
Women	5665			54.3
Smoking				
No, never	4897			48.2
Ex-smoker	2916			28.7
Yes	2356			23.2
Frequency missing = 258				
Physical activity at work				
Mainly seated work	4744			46.9
Main standing/walking work, not strenuous	2425			24.0
Standing/walking work with lifting/carrying or heavy/fast work, physically strenuous	2952			29.2
Frequency missing = 306				
Psychosocial work factors (0–100)				
Emotional demands	10,154	44.6	25.1	
Frequency missing = 273				
Influence at work	10,085	67.4	24.0	
Frequency missing = 342				
Depression				
No	8938			87.5
Yes	1272			12.5
Frequency missing = 217				
Cardiovascular disease				
No	9719			95.2
Yes	489			4.8
Frequency missing = 219				
Cancer				
No	9876			96.8
Yes	331			3.2
Frequency missing = 220				
Low back pain				
Low pain (0–3.9)	7263			71
High pain (4–10)	2964			29
Frequency missing = 200				
Neck-shoulder pain				
Low pain (0–3.9)	7156			70
High pain (4–10)	3064			30
Frequency missing = 207				

Legend: *N* sample number, *BMI* body mass index

Table 2 shows the association between the four types of lifestyle behaviors with low back pain and neck-shoulder pain. The minimally adjusted model (model 1) showed that smoking and being physically active during leisure associated with higher and lower risk of pain in low back, respectively. Also, a daily intake of fruit and vegetables was associated with lower risk of having low back pain in the minimally adjusted model.

**Table 2:** Relative risk (RR) for having pain (≥4 on a scale of 0–10) in the low back and neck-shoulder, respectively, in relation to four types of health behaviors

			Low back pain		Neck-shoulder pain	
			Model 1	Model 2	Model 1	Model 2
	N	%	RR (95% CI)	RR (95% CI)	RR (95% CI)	RR (95% CI)
Alcohol (units per week)						
0–7	5308	52.6	1	1	1	1
8–14	2214	21.9	0.92 (0.85–1.00)	1.00 (0.96–1.03)	0.93 (0.86–1.01)	0.99 (0.92–1.06)
15–21	1270	12.6	0.96 (0.87–1.07)	1.00 (0.95–1.04)	0.93 (0.85–1.03)	0.97 (0.89–1.05)
>21	1306	12.9	1.06 (0.97–1.17)	1.01 (0.97–1.06)	1.03 (0.94–1.14)	1.01 (0.93–1.11)
Physical exercise (hours per week)						
0	1595	15.7	1	1	1	1
1–2	3688	36.4	**0.84 (0.77–0.92)**	0.96 (0.93–1.01)	0.93 (0.85–1.01)	0.97 (0.89–1.04)
3–4	2923	28.8	**0.82 (0.75–0.91)**	0.97 (0.93–1.02)	**0.88 (0.80–0.96)**	0.95 (0.87–1.03)
≥ 5	1938	19.1	**0.83 (0.75–0.93)**	**0.95 (0.90–1.00)**	**0.86 (0.77–0.95)**	**0.90 (0.82–0.99)**
Fruit and vegetable intake (frequency)						
≤ 2/wk	833	8.4	1	1	1	1
3–6/wk	1330	13.4	0.98 (0.86–1.11)	1.01 (0.95–1.07)	1.06 (0.92–1.21)	1.06 (0.94–1.20)
Daily	7734	78.1	**0.87 (0.78–0.97)**	1.00 (0.95–1.05)	1.00 (0.89–1.12)	1.06 (0.95–1.18)
Smoking (cigarettes pr day)						
0	7813	77.3	1	1	1	1
1–9	717	7.1	**1.18 (1.05–1.32)**	1.04 (0.98–1.10)	1.10 (0.98–1.23)	1.03 (0.93–1.14)
10–20	912	9.0	**1.22 (1.11–1.35)**	1.04 (0.99–1.09)	**1.16 (1.05–1.28)**	1.05 (0.96–1.15)
≥ 20	667	6.6	**1.38 (1.24–1.54)**	1.05 (0.99–1.11)	**1.33 (1.19–1.48)**	1.10 (0.99–1.22)

Model 1: Mutually adjusted for the 4 health behaviors + age and gender; Model 2: Mutually adjusted for the 4 health behaviors + age, gender, BMI, physical activity at work, psychosocial work factors (influence and emotional demands), job group, and chronic disease (depression, cardiovascular, cancer)

Significant differences (p<0.05) are marked in bold

In the fully adjusted model (model 2), only a high level of physical activity (i.e. 5 h per week) was associated with lower risk of low back pain and neck-shoulder pain, with RRs of 0.95 (0.90–1.00) and 0.90 (0.82–0.99), respectively.

Estimates for the psychosocial factors that were used as continuous control variables (scale 0–100) are provided here (not discussed): For emotional demands, the OR’s were 1.001 (95% CI 1.001–1.002) for low back pain and 1.003 (95% CI 1.002–1.005) for neck-shoulder pain, i.e. higher emotional demands is associated with higher pain. For influence at work, the OR’s were 0.998 (95% CI 0.998–0.999) for low back pain and 0.997 (95% CI 0.996–0.998) for neck-shoulder pain, i.e. higher influence at work is associated with lower pain.

## Discussion

The present study found that physical activity during leisure was associated with lower risk of having low back pain and neck-shoulder pain when adjusted for age and gender (model 1). Furthermore, when adjusting for age and gender, the level of smoking and physical activity were associated with higher and lower risk of low back pain and neck-shoulder pain, respectively. Daily fruit and vegetable intake was associated with low back pain when adjusted for age and gender, while no association was observed between alcohol intake and pain.

Physical activity is one of the most important characteristics for retaining health [41, 42], and inactivity is considered a major factor for developing various diseases [43]. In addition, there is evidence for using physical activity in the management of chronic low back pain [44]; however, there is still no consistent associations between the studies. Zadro et al. [45] performed a cross-sectional control study with 1588 twins from Spain. The authors evaluated self-reported recent low back pain (within the past 4 weeks), previous low back pain (no pain within the past 4 weeks), and persistent low back pain (no pain-free month in the last 6 months). To evaluate physical activity level, the guidelines of the World Health Organization were used, which recommends at least 75 min of vigorous-intensity physical activity, or at least 150 min of moderate-intensity physical activity, per week. The authors observed an inverse association between recent low back pain and physical activity Conversely, when they controlled the analyses for genetics and shared environment, the association disappeared [45]. However, our findings indicate a lower risk for having low back pain when obeying to the recommendations of the World Health Organization.

Lunde and co-workers [46] performed a follow-up study of 6.5 years in young adults during the transition between school and work life. The authors aimed to investigate the association between low back pain and leisure time physical activity and they did not find trends of reduced low back pain with increased leisure time physical activity. However, the average age in the study was 17.5 ± 1.2 years, while the average age in the present study sample was 43.5 ± 11.7 years. Age could therefore be important since prevalence of low back pain seems to increase with aging [47].

Sitthipornvorakul and co-workers [48] published a systematic review to investigate the associations of physical activity with low back pain and neck pain. Seven studies were included regarding neck pain, however, only one study investigated the associations in a working population. No association was found between physical activity during leisure time and neck pain. Ten high-quality studies were included concerning low back pain, but seven of the studies examined school children. The other three studies investigated a general population and found a relation between low back pain and levels of physical activity.

Another life-style habit that showed assotiations with low back pain and neck-shoulder pain was smoking status, however with adjustment for further covariates no associations were observed in the present study. Smoking could theoretically be a risk factor for developing chronic pain due to the nicotine which leads to an activation of the immune system [49]. The nicotine itself increases the degradation of collagen, decreases blood and oxygen supply, resulting in vascular damage predisposing to back pain, among other conditions [50]. However, the literature still is controversial. Considering low back pain, a cross-sectional study from 2017 investigated 1355 (741 males and 641 females) young Indian administrative service aspirants and medical postgraduate aspirants aged 18–35 years [12]. The results showed no associations between smoking and risk of low back pain. Another cross-sectional study from 2017 [18] investigated 358 male workers from Iran without finding any association between smoking and low back pain.

Both studies [12, 18] are somewhat in line with our findings with smoking not being a risk factor for having low back pain in the fully adjusted model used in this study. However, a study from 2018 [16] based on data of the 2009–2012 National Health Interview Survey (NHIS) of the civilian population of the United States did observe associations between self-reported low back pain and current or former smoking.

To our knowledge, the existing literature on neck-shoulder pain seems to be in accordance with our study results. A study among software engineers observed an association between smoking and current neck pain [51]. Furthermore, a study of 19,665 community residents aged 18–65 years in China observed that the most common complaints of pain among the workers was pain in head, neck/shoulder and back. The study observed, that drinking and smoking status were significantly associated with increased reporting of chronic pain [52].

Considering the other lifestyle factors examined in the present study, neither alcohol nor vegetable and fruit intake showed a consistent association with low back pain and neck-shoulder pain in both the mutually adjusted models. A previous systematic review observed that an association between alcohol consumption and low back pain only appears in complex cases of low back pain and only in people with alcohol consumption addiction [28]. Regarding fruit and vegetable intake, fewer studies were conducted, and in general the evaluation occurred with a grouping analyses, as the study of Skillgate et al. [53]. They included similar healthy lifestyle behavior as the present study (physical activity, alcohol intake, smoking, and diet), and by means of a dichotomization (healthy/not healthy) combined the variables in a categorical variable according to the number of healthy behaviors present. The authors explored the risk of low back pain and neck pain in both genders with the categorical variable and the results showed that a healthy lifestyle behavior seems to be protective for long duration low back pain and neck pain in men and women, respectively. This way of grouping variables is interesting, because pain is multifactorial.

Finally, it is important to note that the wide variation in prevalence of pain could be partially explained by methodological, racial/ethnical, or cultural differences [54]. Still, the differences between the two models used in the present study, with adjustment of covariates, indicate that low back pain and neck-shoulder pain are associated with several factors. Adjusting for several other factors in the second model reduced the association between lifestyle and pain, and only physical activity remained statistically associated with low back pain.

The present study has both limitations and strengths. A limitation of the study is the use of self-reported questionnaires, which may have led to reporting bias. For instance, there could be different understandings and perceptions in people with chronic pain, who often report problems with cognitive abilities, such as memory or attention [55]. However, a questionnaire survey is a relatively low-cost, easy-to-use tool for investigations on health behaviours among the general working population. Another limitation is that the observed results may in part be caused by reverse causation, i.e. people with higher levels of pain may tend to do less physical activity due to the pain. A strength of the study is the methodology used to perform the data analysis, where factors that might interfere with the results such as age, gender, BMI, physical activity at work, psychosocial work factors, job group, and chronic disease were controlled for. However, the control variables for chronic diseases do not consider diseases from respiratory and metabolic systems. Thus, future studies should look specifically into these factors. Another strength is the large sample size, including workers from diverse sectors representing the Danish workers in general, however, only 53% of the invited workers replied the questionnaire. Nevertheless, a previous study performed a robustness analysis that showed that the rating of the working environment was only minimally influenced by the response rate even though the non-response analysis showed that the higher educated job groups had a higher response rate [33].

## Conclusion

Being physically active during leisure associated with lower risk of having musculoskeletal pain, while smoking habits and healthy eating were associated with higher pain when adjusting for age and gender. Considering the relatively high prevalence of unhealthy habits in the general working population, initiatives to promote healthy habits should still be a political priority to help the workers to stay healthy and cope to their work.

## Data Availability

The datasets used and/or analyzed during the current study will be available from Professor Lars L. Andersen on a reasonable request.

## Abbreviations

95% CI: 95% confidence intervals
BMI: Body mass index
CI: Confidence interval
DWECS: The Danish Work Environment Cohort Study
NHIS: National Health Interview Survey
OR: Odds ratio
RR: Risk ratios
